# Genetics and Risk Assessment

**DOI:** 10.2174/138920212802510493

**Published:** 2012-09

**Authors:** H.-U.G. Weier, B. O’Brien

The last decade has witnessed a dramatic evolution in ways genome research is performed including a broader introduction of patient-specific genomic knowledge into the clinical practice. This Hot Topic issue of Current Genomics attempts to capture the breadth of ongoing basic research and matured assays for clinical diagnostic tests. Without claiming to provide a comprehensive overview of current genomics in the field of clinical risk assessment, this compilation of nine mini-reviews prepared by an international group of leading researchers covers a broad range of studies from molecular cytogenetics to bioinformatics and data mining.

Three original contributions focus on the effects of ionizing radiation on DNA, radiosensitivity and genetic instability as predictor of poor prognosis in radiotherapy. The paper by T. Schmid and colleagues (Muenchen, Germany) compares the effects of different radiation qualities in model systems. A review by Greulich-Bode *et al.* (Heidelberg, Germany) focuses on the multifaceted analysis of radiosensitivity. Radiosensitivity is also the theme of a review by C. Rümenapp and collaborators (Muenchen, Germany) concluding that genomic instability can serve as a predictor or surrogate marker for poor prognosis in radiotherapy of sarcomas.

Three diverse papers describes state-of-the-art applications of bioinformatics for risk assessment: the paper by H. Zeng *et al.* (Berkeley, CA) reviews traditional wet-lab and novel data mining-based ways of preparing DNA probes for chromosome enumeration studies. A contribution by V. Sawhney *et al.* (London, UK) elegantly describes potential and promises of genome-wide association studies in cardiovascular research and the hunt for predictive biomarkers. A more matured clinical assay platform for aneuploidy detection combining single cell biopsies of human preimplantation embryos with DNA microarray-based high resolution cytogenetic analyses is reviewed by S. Munné (Livingston, NJ).

One of the remaining three papers in this issue deals with the contribution of current genomics in leukemia research and clinical diagnostics. In their review, K.M. Greulich-Bode and B. Heinze (Heidelberg and Ulm, Germany) discuss the increased role of molecular cytogenetics analyses in blood cancer diagnostics and prognostication in chronic myelogenous leukemia (CML).

Finally, the reader can enjoy a contribution focusing on single-cell genomic analysis of neuronal diversity and neuropsychiatric diseases by I. Iourov and co-investigators (Moscow, Russian Federation). This Hot Topic issue of Current Genomics devoted to genetics, genomics and risk assessment concludes with the description and mini-review of biomarker discovery in the stressed murine central nervous system and marker profiles associated with neuropsychiatric diseases by X. Lowe and A. Wyrobek (Berkeley, CA).

We wish to thank the Editor-in-Chief of Current Genomics, Dr. Christian Neri, for his initiative and stimulation, which lead to the present Hot Topic issue. We express our gratitude to all the authors and reviewers who’s effort have help to shape this issue, and last-but-not-least acknowledge the administrative support from the editorial team at Bentham Publishing Co. Very special thanks go to Dr. Hui Zeng, Berkeley, who superbly managed the correspondence and flow of information between authors and editors.

